# Characterization of *SLITRK1* Variation in Obsessive-Compulsive Disorder

**DOI:** 10.1371/journal.pone.0070376

**Published:** 2013-08-21

**Authors:** Uzoezi Ozomaro, Guiqing Cai, Yuji Kajiwara, Seungtai Yoon, Vladimir Makarov, Richard Delorme, Catalina Betancur, Stephan Ruhrmann, Peter Falkai, Hans Jörgen Grabe, Wolfgang Maier, Michael Wagner, Leonhard Lennertz, Rainald Moessner, Dennis L. Murphy, Joseph D. Buxbaum, Stephan Züchner, Dorothy E. Grice

**Affiliations:** 1 John P. Hussman Institute for Human Genomics, University of Miami Miller School of Medicine, Miami, Florida, United States of America; 2 Department of Neuroscience, University of Miami Miller School of Medicine, Miami, Florida, United States of America; 3 Seaver Autism Center for Research and Treatment, Department of Psychiatry, and the Friedman Brain Institute, Icahn School of Medicine at Mount Sinai, New York, New York, United States of America; 4 Child Psychiatry Department, Robert Debré Hospital, Paris, France & Human Genetics and Cognitive Functions, Institut Pasteur, Paris, France; 5 Institut National de la Santé et de la Recherche Médicale U952, and Centre National de la Recherche Scientifique UMR 7224 and Université Pierre et Marie Curie, Paris, France; 6 Department of Psychiatry and Psychotherapy, University of Cologne, Cologne, Germany; 7 Department of Psychiatry and Psychotherapy, University of Homburg, Homburg, Germany; 8 Department of Psychiatry and Psychotherapy, University of Greifswald, Stralsund, Germany; 9 Department of Psychiatry and Psychotherapy, University of Bonn, Bonn, Germany; 10 Laboratory of Clinical Science, National Institute of Mental Health Intramural Research Program, Bethesda, Maryland, United States of America; 11 Division of Tics, Obsessive-Compulsive and Related Disorders, Department of Psychiatry, and the Friedman Brain Institute, Icahn School of Medicine at Mount Sinai, New York, New York, United States of America; Institute of Psychiatry at the Federal University of Rio de Janeiro, Brazil

## Abstract

Obsessive compulsive disorder (OCD) is a syndrome characterized by recurrent and intrusive thoughts and ritualistic behaviors or mental acts that a person feels compelled to perform. Twin studies, family studies, and segregation analyses provide compelling evidence that OCD has a strong genetic component. The *SLITRK1* gene encodes a developmentally regulated stimulator of neurite outgrowth and previous studies have implicated rare variants in this gene in disorders in the OC spectrum, specifically Tourette syndrome (TS) and trichotillomania (TTM). The objective of the current study was to evaluate rare genetic variation in *SLITRK1* in risk for OCD and to functionally characterize associated coding variants. We sequenced *SLITRK1* coding exons in 381 individuals with OCD as well as in 356 control samples and identified three novel variants in seven individuals. We found that the combined mutation load in OCD relative to controls was significant (*p* = 0.036). We identified a missense N400I change in an individual with OCD, which was not found in more than 1000 control samples (P<0.05). In addition, we showed the the N400I variant failed to enhance neurite outgrowth in primary neuronal cultures, in contrast to wildtype *SLITRK1*, which enhanced neurite outgrowth in this assay. These important functional differences in the N400I variant, as compared to the wildtype *SLITRK1* sequence, may contribute to OCD and OC spectrum symptoms. A synonymous L63L change identified in an individual with OCD and an additional missense change, T418S, was found in four individuals with OCD and in one individual without an OCD spectrum disorder. Examination of additional samples will help assess the role of rare *SLITRK1* variation in OCD and in related psychiatric illness.

## Introduction

Obsessive-compulsive disorder (OCD) is characterized by recurrent and intrusive thoughts (obsessions) and ritualistic behaviors or mental acts (compulsions) that are frequently performed in response to the obsessional thoughts [1]. Twin and family studies, the latter including segregation analyses, have provided compelling evidence that OCD has a strong genetic component with a complex genetic architecture [2]. It is likely that OCD is etiologically quite heterogeneous and that rare variants may account for an appreciable proportion of the genetic liability to OCD. Family and treatment studies indicate that Tourette syndrome (TS) and trichotillomania (TTM) are related to OCD and all three conditions might be considered part of a larger spectrum of conditions, the so-called OC spectrum [1], [3].

 Rare variants in the gene *SLITRK1* have been associated with disorders in the OC spectrum [4]–[6]. In the first description, genetic analysis of a subject with TS revealed a *de novo* translocation in 13q implicating *SLITRK1* as the etiologic mutation [4]. The same study also described a frameshift mutation in *SLITRK1* in an additional subject with TS, and a recurrent variant in the 3′ UTR associated with altered microRNA binding [4], [7]. The frameshift mutation in the TS proband was inherited from a mother with TTM. Subsequently, an independent screening of TTM individuals for mutations in *SLITRK1* found two additional missense mutations that were significantly associated with TTM [5].

*SLITRK1* is a member of a family of structurally related transmembrane proteins characterized by leucine-rich repeat domains in the extracellular domain and functionally divergent cytoplasmic domains [8]. The cytoplasmic domain of *SLITRK1* distinguishes it from the other members of the *SLITRK* protein family (*SLITRK 2-6*), which may explain its differing role in neurite outgrowth. *SLITRK1* promotes neurite outgrowth while the other *SLITRK* proteins tend to inhibit neurite outgrowth [8]. Animal studies demonstrate that a *SLITRK1* knockout mouse has an anxiety-like phenotype that responds to alpha adrenergic compounds akin to pharmacological treatments of the human OC spectrum while a knockout model of a related gene, *SLITRK5*, results in mice that exhibit OCD-like behaviors, including anxiety and excessive-self grooming [9], [10].

In the current study we investigated the occurrence of rare variation in *SLITRK1* individuals with OCD using a case-control association methods. We present the results of a genetic screen of *SLITRK1* in OCD and in ethnically matched controls screened for the absence of OCD (specifically OCD, TS, and TTM). Using two primary neuronal *in vitro* model systems we then describe the functional impact of a rare coding variant identified in the screen.

## Results

### Genetic Screen

The complete coding region of *SLITRK1* was sequenced in 381 unrelated Caucasian individuals with OCD and 356 ethnically matched controls screened for the absence of disorders of the OC spectrum (OCD, TS, and TTM) [11]. Additional control samples used for N4001 genotyping (n = 679) were also matched for ancestry and had been screened for the absence of OCD and related disorders [25].

**Figure 1:**
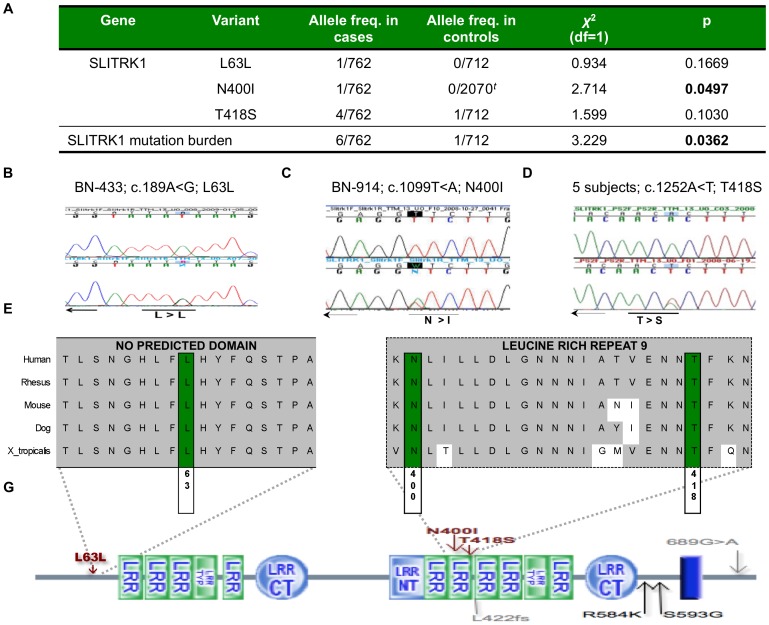
Novel mutations identified in *SLITRK1*. (**a**) Allelic frequency of novel *SLITRK1* mutations identified in obsessive-compulsive disorder (OCD) cases. (**b**) The *SLITRK1* L63L mutation has been demonstrated by sequencing in one of 762 OC spectrum alleles and in zero of 712 control alleles. (**c**) The *SLITRK1* N400I mutation has been demonstrated via sequencing and genotyping in one of 646 OC spectrum alleles and in zero of 2070 control alleles, respectively; *^t^*includes genotyping of 1358 alleles. (**d**) The *SLITRK1* T418S mutation has been demonstrated by sequencing in three of 762 OC spectrum alleles and in one of 410 control alleles. (**d**) Conservation map of *SLITRK1* region where the three novel mutations were identified (Green – novel variants, gray – evolutionarily conserved regions). (**e**) A schematic of the *SLITRK1* protein with the detected variants identified (red – novel variants, gray – previously published variants in Tourette syndrome^4^, black – published variants in trichotillomania^5^. Dotted outline depicts leucine rich repeat (LRR) region 9. Diagram of *SLITRK1* is available at http://smart.embl-heidelberg.de. LRR typ – LRR typical subfamily, LRR CT – LRR C-terminal domain, LRR N-terminal domain; dark blue bar – transmembrane domain.

We identified three novel changes in *SLITRK1*, L63L (c.189A<G), N400I (c.1099T<A) and T418S (c.1252T<A). Two of these (L63L and N400I) were identified only once, in both cases in a subject with OCD; both subjects were heterozygous for the change. We identified the third heterozygous nonsynonymous change, T418S, in four individuals with OCD and in one non-OC spectrum control. Each of the identified variants was found in a highly conserved amino acid (Figure 1).

The combined mutation load for *SLITRK1* in OCD (1.57% of cases) compared to controls (0.28% of controls) was significant (chi-square statistic (*χ* ^2^) = 3.229, df = 1, *p* = 0.036). Genotyping did not identify the *SLITRK1*-N400I variant in 679 additional controls (*χ* ^2^ = 2.714, df = 1, *p* = 0.0497). In addition, comprehensive review of the data release from the three 1000 Genomes Project pilots did not identify the N400I variant in over 1000 low coverage genomes and whole exomes. [Variants identified in the 1000 Genomes Project included S49N, S330A, N358I, L380I, T148S, M507V, and K541R. All were identified in one sample, with the exception of S330A, which was identified in 9 samples. None of these variants was observed in our study.]

### Phenotypes

The *SLITRK1*-L63L change was found in one individual with OCD. She was diagnosed with OCD at age 33, never achieving full remission while in treatment. Comorbid major depressive disorder (MDD), social phobia, and post-traumatic stress disorder (PTSD) were present. She did not have TTM, TS or any other tic disorder; however, the family history for these disorders is unknown.

The individual in whom we identified the N400I mutation had OCD diagnosed at age 47 (age of onset of clinical symptoms is not clear) and never fully remitted. The individual presented with obsessive rituals that responded to treatment with amitriptyline, and later clomipramine. He did not have TTM, TS or any other tic disorder. Family history for these disorders is unknown.

**Figure 2:**
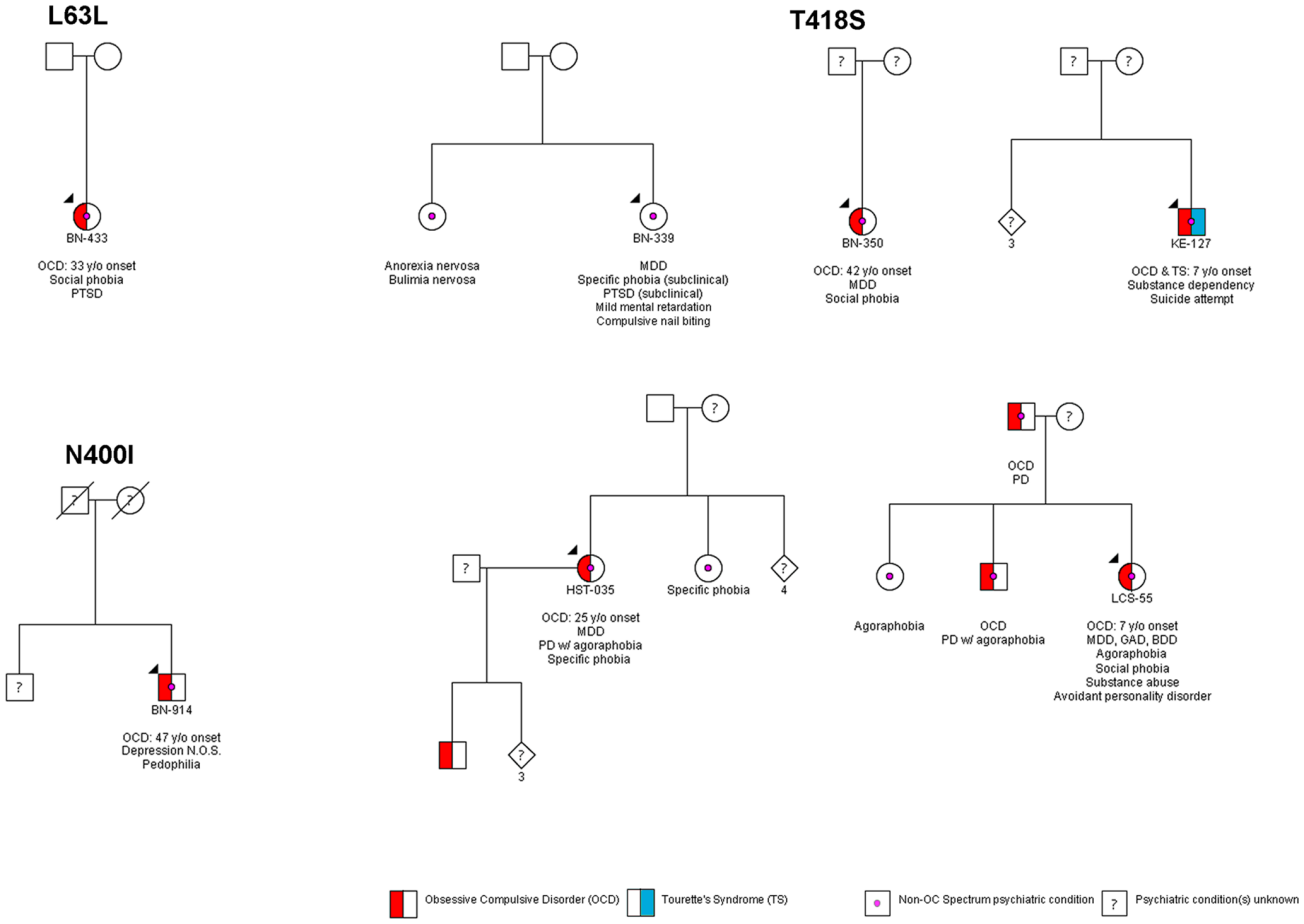
Pedigree diagrams of families with *SLITRK1* variants. Pedigrees for individuals in whom *SLITRK1* variants were identified. Each obsessive-compulsive (OC) spectrum proband is labeled with his/her identifier and is designated by a black arrowhead. Individuals affected with an OC spectrum disorder are represented by shaded symbols, with red shading indicating obsessive-compulsive disorder (OCD) and blue shading indicating Tourette syndrome (TS). Psychiatric conditions outside of the OC spectrum are represented by a magenta circle in the center of the symbol. Male family members are represented with squares, females with circles, persons with unspecified gender are diamonds with the number of individuals indicated directly below. All psychiatric pathology is listed under each affected individual. OCD – Obsessive-Compulsive Disorder, TS – Tourette syndrome, BDD – Body Dysmorphic Disorder, GAD – Generalized Anxiety Disorder, MDD – Major Depressive Disorder, N.O.S. – not otherwise specified, PD – Panic Disorder, PTSD – Post-Traumatic Stress Disorder, w/ - with, y/o – years old, ? – psychiatric history is unavailable for the individual.

The *SLITRK1*-T418S change was found in four individuals with OCD and in one ethnically matched control. Of the individuals affected with OCD, one with the T418S mutation had onset of OCD at 25 years of age. She also had comorbid MDD, panic disorder with agoraphobia and specific phobia. The second individual with the T418S mutation had onset of OCD at seven years of age. In the same year he was also diagnosed with TS. Neither the TS nor the OCD remitted. The third individual with the T418S mutation had onset of OCD at six years of age with no additional comorbidities known. The fourth individual with the T418S variant had onset of OCD at age 42. She had comorbid MDD, social phobia, and social anxiety disorder. She did not have TTM, TS or any other tic disorder. *Post hoc* analysis of the ethnically matched control identified with the T418S change indicated the presence of psychiatric conditions outside of the OC spectrum, including a grooming disorder (compulsive nail-biting) and MDD. Pedigrees are given in Figure 2.

### Functional Analyses

**Figure 3:**
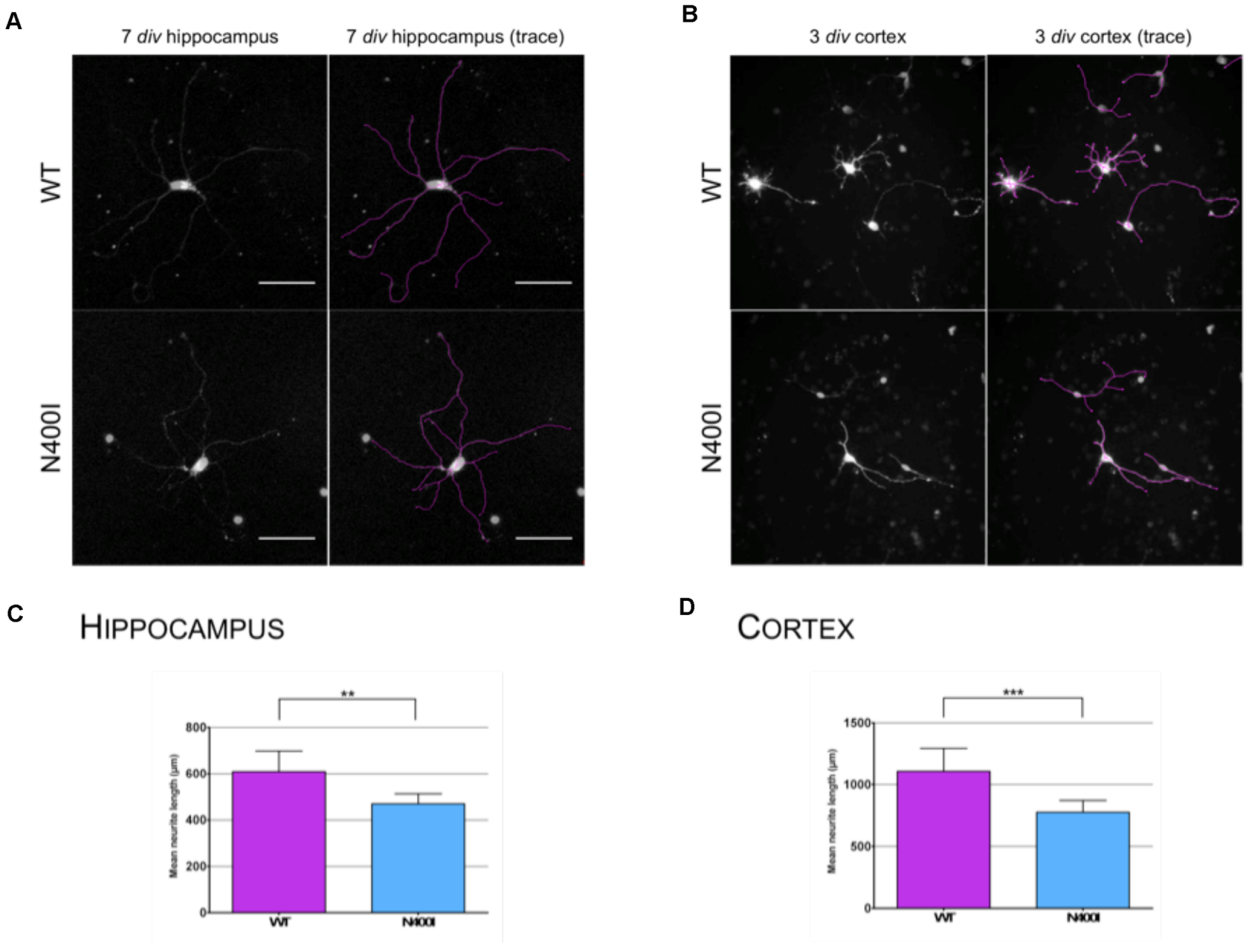
*SLITRK1* variant N400I fails to induce neurite outgrowth. (**a**) LS: Representative images of primary rat E18 hippocampal neurons nucleofected with wildtype *SLITRK1* or the *SLITRK1*–N400I variant. RS: Images are also traced to facilitate visualization of thin neurites. Scale bar = 50 µm (**b**) LS: Representative images of primary mouse E17 cortical neurons nucleofected with wildtype *SLITRK1* or the *SLITRK1*–N400I variant. RS: Representative E17 cortical neuron trace. Scale bar = 50 µm (**c**) The summed total neurite length per hippocampal neuron at 7 *div* is shown. Each bar on the bar graph represents pooled data of at least 50 neurons per experiment (*n* = 3). Images are uniformly overexposed to improve neurite visibility. (**d**) The summed total neurite length per cortical neuron at 3 *div* is shown. Each bar on the bar graph represents pooled data of at least 14–22 neurons per experiment (*n* = 2). Statistical significance was assessed using a student's t-test as described under Materials and Methods. Error bars are 95% confidence intervals. * *p*<0.05, ** *p*<0.01, *** *p*<0.001.

The nonsynonomous changes in *SLITRK1* observed in our study could potentially impact protein function in deleterious ways. *SLITRK1* has been shown to stimulate neurite outgrowth [8], [12] and we therefore assessed the change in neurite length in primary rat neurons transfected with various *SLITRK1* constructs. In E18 rat hippocampal neurons, over-expressed wildtype *SLITRK1* stimulated neurite outgrowth to a significantly greater extent compared to the over-expressed N400I mutant and empty control vector at 7 days in vitro (*div*) (data not shown). At 7 *div* an independent student's t-test demonstrates a significant difference in the summed total neurite length for wildtype (geometric mean (M) = 1377.21 µm, 95% confidence interval [CI_.95_] = 1210.60–1566.75) relative to the N400I condition (M = 1104.08 µm, CI_.95_ = 990.83–1233.11); t(112) = 2.637, *p* = 0.0096 (Figure 3a,c). An additional study of E17 mouse cortical neurons produced similar results. Specifically, our analyses demonstrated significantly longer average neurites at 3 *div* in the *SLITRK1* wildtype (M = 1113.9 µm, CI_.95_ = 992.9–1234.9) compared to the N400I variant (M = 735.4 µm, CI_.95_ = 665.2–805.5); t(72) = 5.564, *p*<0.0001 (Figure 3b,d).

## Discussion

By sequencing coding exons of SLITRK1 in 381 individuals with OCD and 356 controls, we identified two novel missense variants and one novel synonymous variant in *SLITRK1* in seven individuals. Six of the individuals had OCD (1.57% of OCD) and one individual was in the comparison group and did not have an OC spectrum disorder (0.30% of controls). The N400I and L63L variants were identified, each in a separate OCD individual. The T418S variant was identified in four OCD samples and in one screened control. The *SLITRK1* mutation burden in OCD cases relative to controls was significant at *p* = 0.036. This finding highlights the potentially complex nature of *SLITRK1*'s influence on the heritability and expression for disorders in the OC spectrum. Previous studies have demonstrated a significant association between *SLITRK1* and TS and TTM, but a number of independent studies report difficulty in replicating the findings [4]–[7], [13]–[21]. Here we add to the literature three additional variants, one of which (the N400I variant), occurs more frequently in cases than in controls (*p* = 0.0497) (Figure 1a).

Given the high detection frequency of the T418S variant and its discovery in an individual without an OCD, we reasoned that the variant might be common and functionally tolerated in the general population. Similarly, the L63L variant identified in an individual with OCD is unlikely to demonstrate easily identifiable functional consequences, as it does not cause a coding change in the protein. It remains possible that the L63L variant could have subtle functional effects such as altering mRNA folding, decreasing mRNA translation or impacting posttranslational modifications of the protein, consistent with literature that demonstrates functional effects of synonymous mutations in the schizophrenia candidate gene, human dopamine receptor D2 (DRD2) [22]. Thus, while the T418S and L63L variants may have a role in increasing susceptibility to an OC spectrum disorder, it may be hard to demonstrate functional impact of these variants with in vitro functional assays.

In contrast, the *SLITRK1*-N400I variant is a missense change that in theory carries a greater risk for secondary functional consequences and phenotypic differences. Our functional analysis of the N400I variant in rat hippocampal neurons and in mouse cortical neurons supports this conclusion. Wildtype *SLITRK1* stimulates neurite outgrowth [4], [12] and we investigated the impact of the N400I variant on this function. Unlike the overexpressed wildtype *SLITRK1*, which significantly extended the summed total neurite length in our neurite outgrowth assays (relative to an empty vector control, data not shown), the N400I variant showed no activity in either E18 rat hippocampal neurons nor in E17 mouse cortical neurons. Similarly, the *SLTRK1* frameshift mutation previously observed in TS and TTM cases also failed to stimulate neurite outgrowth [4]. The consistent functional alteration seen in both the *SLITRK1* frameshift variant and in the N400I variant provides support for a role for the N400I variant in the pathophysiology in the OC spectrum. More specifically, the amino acid change in N400I is located in one of the extracellular leucine-rich repeat domains (a type of domain predicted to be involved in protein-binding) and as such may disrupt the interaction of *SLITRK1* with its extracellular targets, leading to inhibited or compromised function.

We identified the *SLITRK1*-T418S missense variation in four individuals with an OCD and in one individual without an OC spectrum disorder. *Post hoc* analysis revealed that the individual without an OC spectrum disorder is afflicted with MDD, is mildly mentally retarded and exhibits various subclinical psychiatric phenotypes, despite the absence of an OC spectrum disorder (Figure 2). In addition, she exhibits a pathological grooming behavior, compulsive-nail biting, that is reminiscent of similar grooming behaviors seen in the OC spectrum, such as compulsive hair-pulling in TTM. Additionally, we note a potential relationship between the *SLITRK1*-T418S variant in depression as four of the five individuals with the T418S missense mutation (including the non-OC spectrum individual) have a diagnosis of MDD and the remaining individual has a history of attempted suicide (Figure 2). However, given the high prevalence rates of depression in the general population (9.1% current depression in the United States [23]), the depressive symptoms and T418S connection seen in this sample may be coincidental. Additional sequencing of *SLITRK1* in large numbers of individuals will be needed to determine whether the T418S or other rare variants, are increased in a broader range of psychiatric phenotypes than is represented by the OC spectrum.

In this study we combine a comprehensive genetic screen of *SLITRK1* in OCD and closely related disorders, with the functional analysis of a newly identified *SLITRK1* missense variant found in the screen. Using this dual approach, we documented significantly more genetic variation in *SLITRK1* in OCD than in a non-OC spectrum comparison group; furthermore, we identified one variant, *SLITRK1*-N400I, which eliminates a known function of the protein. This represents an important advance in the characterization of the role *SLITRK1* may play in the pathophysiology of the OC spectrum and emphasizes the benefits of combining genetic and functional assays to enhance our understanding of psychiatric illness.

## Materials and Methods

### Ethics Statement

This research was approved by the Ethics Committee of the Medical Faculty, University of Bonn Biomedical Center. Informed written consent was obtained from each participant. The consent process was overseen and documented in accordance with the local Research Ethics Boards. All participants who declined to participate or did not otherwise participate were not disadvantaged in any way by not participating in the study. This investigation was conducted according to the principles expressed in the Declaration of Helsinki.

All studies involving animals were approved by local Research Ethics Boards/IACUC at the relevant sites (Miller School of Medicine University of Miami, Icahn School of Medicine at Mount Sinai) and conducted according to the relevant national and international guidelines.

### Subjects

The OCD sample was derived from a large European family study of OCD [11]. Individuals were screened for OCD symptoms (e.g., checking, washing, need for symmetry, obsessions, etc) via a self-rating instrument. Individuals with scores >95^th^ percentile were selected for direct assessment. Affected individuals were diagnosed with OCD using direct interviews performed by trained clinicians (psychiatrists, psychologists or doctoral level clinicians) who completed reliability training. Diagnostic instruments used to screen subjects and controls included the German version of the Schedule for Affective Disorders and Schizophrenia-Lifetime Version, Modified for the Study of Anxiety Disorders (SADS-LA-IV) and/or the Structured Clinical Interview for DSM IV and, for subjects with OCD, additionally, the Family Informant Schedule and Criteria (FISC) with additional sections for detailed assessment of OCD (i.e., YBOCS), tics, TS and related disorders. All diagnoses were made according to DSM IV criteria, using consensus diagnosis techniques. Additional details of subject inclusion and assessments are available [11].

### Sequencing

Genomic DNA was extracted from peripheral blood samples using standard procedures. An optimized PCR cycling protocol was used to amplify the entire *SLITRK1* coding region (available on request). PCR product purification was completed with QuickStep™2 SOPE resin (Edge BioSystems, Gaithersburg, MD). The entire coding sequence of *SLITRK1* was directly sequenced using the Applied Biosystems (ABI, Foster City, CA, USA) BigDye® Dye Terminator Cycle Sequencing Kit on an ABI 3730 capillary sequencer. Primers used for amplifying the full coding length of *SLITRK1* are available upon request. Sequences were analyzed with Sequencher software (ver. 4.8, Gene Codes Corp, Ann Arbor, Michigan, USA). Variants in *SLITRK1* were confirmed with re-sequencing fresh DNA aliquots in both directions.

### Creation of constructs

The dsRed-tagged wildtype human *SLITRK1* expression vector used in the hippocampal assay was made by subcloning the human *SLITRK1* cDNA clone (Santa Cruz Biotechnologies, SCBT, 4816570) into a dsRed N1-Express vector (Clontech, Mountain View, CA). The green fluorescent protein (GFP)-tagged wildtype human *SLITRK1* expression vector used in the cortical neuron analyses has been described elsewhere [12]. The N400I mutation was introduced into both vectors by PCR-based mutagenesis and confirmed by sequencing.

### Neurite outgrowth assays

The neurite outgrowth assay was performed on E17 mouse embryo cortical neurons as previously described [12]. Briefly, cortical neurons were prepared from E17 mouse embryo and hippocampal neurons were prepared from E18 rat embryo, and the empty vector, *SLITRK1* wildtype or N400I mutant vector were nucleofected along with GFP, or the dsRed expression vector (cortical and hippocampal neurons, respectively). Three days in vitro (*div*) cortical cultures were fixed by 4% formaldehyde and fluorescent images of neurons expressing GFP were randomly captured. Neurite length was analyzed using Neurolucida (MBF Bioscience, Williston, VT). At least 14 neurons were analyzed for each group. Seven *div* E18 rat hippocampal cultures were fixed by 4% formaldehyde and fluorescent images were selected by a person blind to the treatment condition. Only neurons with pyramidal morphology and unobstructed neurites were analyzed. At least 50 dsRed positive neurons within each condition were reconstructed on a confocal microscope (Zeiss LSM 710 microscope, 40× oil objective) and traced with the Simple Neuron Tracer ImageJ plugin [24].

### Statistics

Chi-square statistics to compare the sequencing and genotyping results in individuals with OCD and controls were generated on contingency tables using GraphPad Prism version 5.00 for Windows (GraphPad Software, San Diego, California, USA). Statistical analysis on quantitative neurite data was carried out using independent student's t-tests with a significance level of *p*<0.05 using PAWS/SPSS Statistics 18.0 software (IBM Corporation, Somers, NY, USA) and/or GraphPad Prism 5.00. The hippocampal summed total neurite length data was non-normally distributed and these data were transformed using a logarithmic transformation. All reported data (excluding t-values) are back-transformed unless otherwise stated.
